# Advancing Circular Bioeconomy through a Systems-Level Assessment of Food Waste and Industrial Sludge Codigestion

**DOI:** 10.1021/acsenvironau.5c00049

**Published:** 2025-07-01

**Authors:** Md. Nizam Uddin, Cassidy Hartog, Emma Murray, Jacob B. Loveless, Lukas Roberson, Asli Aslan, Francisco Cubas, Lewis S. Rowles

**Affiliations:** † Department of Civil Engineering and Construction, 7604 Georgia Southern University, Statesboro, Georgia 30458, United States; ‡ Math Department, Georgia Southern University, Statesboro, Georgia 30458, United States; § Institute for Water and Health, Georgia Southern University, Savannah, Georgia 31419, United States; ∥ Department of Biostatistics, Epidemiology, and Environmental Health Sciences, Georgia Southern University, Statesboro, Georgia 30458, United States

**Keywords:** anaerobic codigestion, microbial community dynamics, life cycle assessment, techno-economic analysis, organic waste valorization, industrial symbiosis, sustainable waste management

## Abstract

Disposal of food waste (FW) in landfills remains an unsustainable practice for organic waste management. Simultaneously, pulp and paper mills produce significant amounts of recalcitrant organic waste that is difficult to decompose due to its high lignocellulosic content. In this study, we developed an innovative approach to improve the digestion of pulp and paper mill sludge (PPMS) by amending FW to produce a low chemical oxygen demand (COD) sludge while recovering methane in the process. This codigestion process was evaluated through lab-scale biogas production experiments coupled with a comprehensive economic and environmental sustainability assessment. Biomethane production results revealed that the FW-PPMS codigestion methane yield was 36% higher on average than the PPMS monodigestion. Additionally, metagenomic analysis revealed that microbial communities for both systems transitioned from highly heterogeneous to more adapted uniform communities after digestion. Improved microbial communities contributed to higher COD removal (92%) in the FW-PPMS system compared to monodigestion (80% removal). The sustainability analysis revealed that the codigestion of FW-PPMS had median costs of 236.64 USD·tonne^–1^·day^–1^ and emissions of 228.30 kg CO_2_ eq·tonne^–1^·day^–1^, a significant reduction compared to directly disposing the FW in landfills (median costs of 405.13 USD·tonne^–1^·day^–1^ and emissions of 556.27 kg CO_2_ eq·tonne^–1^·day^–1^). A nationwide contextual analysis revealed that out of six regions, the US Northeast had the lowest median costs and emissions, while the Mountain Plains region had the highest, highlighting the importance of geographical and infrastructural factors in implementation. Overall, codigesting FW with PPMS is revealed to be a sustainable waste management option to decrease landfill disposal of valuable organic waste.

## Graphical abstract

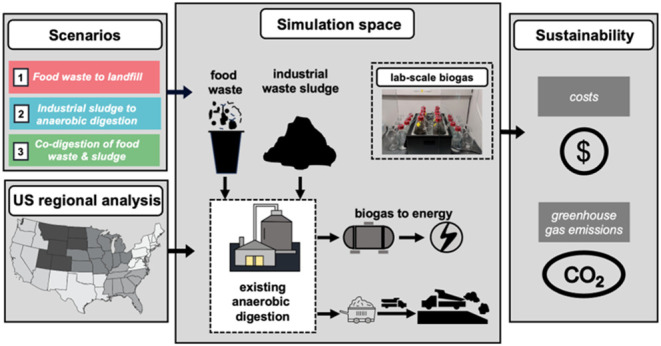

## 1 Introduction

Food waste (FW) is a major contributor (∼24%) to municipal solid waste, and its disposal at landfills has significant environmental and economic impacts.^1,2^ For example, 103 million tons of food were wasted in the US in 2018.^3^ This waste significantly affects greenhouse gas emissions, with FW accounting for 58% of methane emissions from US landfills in 2020; thereby exacerbating global warming and environmental degradation.^4^ Universities are among the largest FW generators in the US, with their dining halls and campus eateries generating an estimated 100–142 pounds of food waste per student annually.^3,5–7^ Recognizing these issues, some states (e.g., California and New York) have passed bills to recycle organic waste at its source. This approach aims to decrease the amount of organic material sent to landfills. Anaerobic digestion (AD) has emerged as a promising solution for FW treatment, offering multiple benefits, e.g., renewable energy production, reduced greenhouse gas emissions, and the generation of nutrient-rich digestate for agricultural use.^8–10^ This technology presents a particularly attractive option for universities and other institutions having similar waste problems (e.g., schools, army bases, etc.) seeking to manage their FW more sustainably. However, AD of FW alone faces several obstacles, including the accumulation of volatile fatty acids (VFAs), process instability, low buffer capacity, and high construction and operation costs.^11,12^ While AD presents a pathway toward more sustainable waste management, these challenges highlight the need for innovative approaches to optimize the process, especially for large institutional waste generators like universities. Therefore, developing synergistic opportunities for AD systems to handle the FW sustainably generated in daily life is needed.

Another major source of organic waste is the pulp and paper mill industry, which is a common industry in the US (Figure S1). Organic waste is generated mainly during the treatment of their effluent water. Approximately 50 kg of dry sludge per tonne of paper is produced in an aerated stabilization basin, which in many cases is the treatment process if not proceeded by AD.^13^ Pulp and paper mill sludge (PPMS) contains a large portion of lignocellulosic material (∼50%), which is difficult to decompose.^14–16^ Also, inhibitors like resin acids, sulfur, and long-chain fatty acids hinder the overall digestion process.^17,18^ These inhibitory factors and recalcitrant materials in PPMS significantly impair the AD process, reducing methane yields and resulting in inadequate sludge stabilization. Consequently, the digestion efficiency is often suboptimal, and the resulting digestate may not meet the desired quality standards for beneficial use or disposal. Due to the difficulties faced in treating PPMS in AD, pretreatment of the sludge and codigestion with a second substrate can be possible options to improve the overall digestion process. Pretreatment through enzyme (e.g., cellulases, hemicellulases, ligninases) addition and physical, chemical, and biological methods has been shown to improve the overall digestion process and increase methane yield, but it may require a substantial capital cost investment to implement at a large scale.^19,20^ Codigestion offers several benefits, including improved nutrient balance (in terms of C/N ratio and macro- and micronutrient abundance) and reduction of inhibitors and toxic compounds.^21–24^ For example, the lab-scale codigestion of PPMS and FW has been shown to yield high methane production (432.3 mL/g VS fed) and a maximum soluble chemical oxygen demand (COD) removal efficiency (87%).^25^ The high nutrient content and low toxicity of FW can be used to overcome nutrient deficiency by improving the carbon-nutrient ratio of PPMS. Due to its high energy and moisture content, FW can be an ideal candidate for AD.^26–28^ Also, apart from a handful of research,^29–31^ a critical gap exists in our mechanistic understanding of the microbial communities driving biogas production in codigestion systems utilizing FW and PPMS. Comprehensive studies elucidating the complex microbial interactions, metabolic pathways, and community dynamics in these integrated systems are notably scarce. This lack of in-depth microbiological insight hinders our ability to optimize codigestion processes and predict system performance across different operational contexts, and ultimately impedes the wider adoption and scalability of these promising waste management practices.

Sustainable assessment tools, such as techno-economic analysis (TEA) and life cycle assessment (LCA), have been employed to analyze the relative sustainability of individual AD feedstock, e.g., wastewater,^32,33^ food waste,^34^ and paper mill waste.^35–37^ For the codigestion of diverse organic feedstocks, multiple benefits have been elucidated using these sustainability assessment tools, including enhanced biogas production, facilitation of microbial synergistic effects, improved nutrient balance, and mitigation of inhibitor concentration.^38,39^ Specifically, experimental codigestion of sugar cane scum with agricultural crop residues increased methane yield by 30.2 and 5.9% compared to their respective monodigestion processes.^40^ Additionally, the codigestion of wet hydrolyzed dissolved air flotation sludge and stockyard reported dilution of inhibitory compounds, e.g., the residual toxic hexane and higher methene yield from the codigested system.^41^ Despite these advantages, an understanding of the economic and environmental implications of codigestion remains limited, with only a handful of studies suggesting that it may lead to lower costs and emissions.^42,43^ To fully comprehend the potential of codigestion of FW and PPMS, a comprehensive end-to-end analysis is needed, considering appropriate contextual parameters and variables. This analysis should encompass all stages of the process—from waste generation and collection through transportation and AD to final disposal, integrating both TEA and LCA methodologies to provide a comprehensive sustainability assessment.

The overall goal of this research was to understand the potential of codigesting FW with PPMS by leveraging the current paper mill infrastructure. Specifically, the objectives of this work were (i) to gain a mechanistic understanding of the digestibility of PPMS and FW through lab-scale studies and (ii) to estimate the costs and global warming potential (GWP) of this codigestion process at scale. To assess these objectives, bench-scale anaerobic digesters were operated with a 1:1 sludge-to-inoculum ratio (VS basis), monitoring biomethane potential over time along with key operational parameters [VS, total solids (TS), alkalinity, VFA, chemical oxygen demand (COD), ammonia, pH, total organic carbon (TOC), and total nitrogen (TN)]. For sustainability analysis, we modeled three scenarios in Python: (i) FW disposal to the landfill, (ii) PPMS treatment with end-of-life landfill disposal, and (iii) combined FW-PPMS treatment with landfill disposal. These scenarios were selected to provide the status quo (scenarios (i) and (ii)) and an integrated approach (scenario (iii)). System drivers were identified through uncertainty analysis using Monte Carlo simulation and sensitivity analysis using Spearman’s rank correlation coefficients. The analysis also examined trade-offs among these scenarios across six geographical regions of the US by adjusting context-specific variables. A better understanding of codigestion mechanisms and limitations will enable better prediction, optimization, and control of biogas production as well as process stability. This comprehensive approach allows us to navigate the complex landscape of technology development pathways and provide insights to inform decision-making for more sustainable waste management practices.

## 2 Materials and Methods

### 2.1 Lab-Scale Studies to Assess the Feasibility of Codigestion

#### 2.1.1 Feedstocks and Inoculum

PPMS was obtained from a local paper making facility in Georgia. The sample was collected from one of the treatment ponds, an intermediate aerated treatment stage, and stored frozen (−8 °C) until experimental use. FW, consisting of fresh onions, tomatoes, and blueberries, were bought from a local Walmart Store in Statesboro, Georgia. FW ingredients were selected based on the higher biochemical methane potential yield to experiment with ideal conditions.^44^ The objective was to guarantee quantifiable biogas production for analysis; however, it is possible that lower yields would have resulted with different substrates. These ingredients were equally weighed and blended to produce the mix. Next, the resulting mix was sieved through a 2.74 mm mesh to ensure homogeneity. A sludge inoculum, which was part of the final mix, was collected from the South Columbus Water Resource Facility located in Columbus, Georgia. It was shipped overnight and, upon collection, incubated at 35 °C for approximately 60 h before starting the biogas experiments. This incubation period aimed to degas the inoculum and mitigate its impact on the experimental methane production.^45^ If the inoculum contributes more than 20% of the total methane yield, incubation becomes necessary.^46^ This approach ensures an accurate assessment of the substrate’s methane potential without significant interference from the inoculum.

#### 2.1.2 Biomethane Potential Experiments

Bench-scale experiments were carried out using a sludge-to-inoculum ratio of 1:1 on a VS basis. 1000 mL sterile GL45 bottles served as reactors with 600 mL working volume. The VS of the inoculum and the substrates were measured initially and then placed in the reactor bottles maintaining the proper ratio. The rest of the volume was filled by sterile deionized water. The initial FW pH was adjusted to 7.0 by using a NaOH solution. In addition to FW and PPMS codigestion, reactors were set up for PPMS monodigestion and a blank (inoculum only) as a control. FW monodigestion was not conducted as the research compared the existing treatment practices with the proposed codigestion approach, i.e., whether the addition of FW improves the methane yield or not in the codigestion system. Codigested reactors were run in quadruplicate, where the monodigested and blank were run in triplicate. To ensure anaerobic conditions, the reactors’ headspace was flushed with pure nitrogen gas for 3 min and immediately sealed with stainless steel caps.^47^ Reactors were then placed in a thermostatically controlled water bath at 35 ± 2 °C for temperature stability. Each reactor was connected to a bottle with a 3N NaOH solution to absorb the carbon dioxide produced. Finally, the produced methane volume was measured by NaOH solution displacement into an empty bottle.^47^ The reactors were mixed vigorously daily to ensure a homogeneous environment. The reactors were run for 31 days, until they stopped producing gases. Specific methane yield from PPMS and FW-PPMS digesters was calculated by subtracting the total methane from the blank digesters. The resulting volume was then adjusted to an equivalent volume at a standard temperature and pressure (STP). Then, the adjusted total volume (mL) was divided by the VS (g) mass added in each digester (Section S1).

#### 2.1.3 Characterization Methods

PPMS, FW, and inoculum were characterized at the beginning and end of the experimental setup. TS and VS were determined using standard method 2540.^48^ pH was measured using an F20 (Toledo) pH meter. Sludge samples were diluted (three times for the pre, no dilution for the post) with deionized water and centrifuged at 4500 rpm for 15 min. COD, VFA, and TAN of the supernatant were measured using a Hach DR1900 spectrophotometer following TNT methods 822, 872, and 830, respectively. Supernatant samples were further analyzed for total organic carbon (TOC) and total nitrogen (TN) using a total organic carbon analyzer (TOC-L, Shimadzu, Japan) coupled to a total nitrogen module (TNM-L). For all of the methods, the calculated values were well under the detection limits.

#### 2.1.4 Analysis of Microbial Community

Genomic DNA was isolated from 25 digestion samples using the DNeasy PowerSoil Pro Kit (Qiagen, Germany). The 16S rRNA gene was amplified using long-read 341-1492 PCR primers,^49–51^ followed by SMRTbell library preparation and sequencing on the PacBio Sequel platform at MR DNA (Molecular Research LP). After quality filtering and processing using the MR DNA analysis pipeline, operational taxonomic units (OTUs) were defined by clustering at 97% similarity^52,53^ and taxonomically classified using BLASTn against the NCBI database.^54^

A total of 224 genera were identified across all of the samples. PRIMER 7 (v7.0.23)^55^ was used for multivariate analysis comparing genera counts among pre- and post-digestions. Genera accounting for ≥1% of the total in each digestion were considered abundant.^56,57^ For abundant genera, their ability to utilize different carbohydrates and proteins was assessed. One-way permutational multivariate analysis of variance (PERMANOVA) was used with digestion as the factor to compare genera counts, and nonmetric multidimensional scaling (NMDS) was used for visualization. Diversity metrics (Shannon-Weiner, Simpson) were calculated using RStudio (v2024.04.02).^58^ (Detailed DNA extraction, PCR amplification, and sequencing protocols are provided in Supporting Information Section S2).

### 2.2 Sustainable Design of Integrated Codigestion

#### 2.2.1 Overview of Collection, Treatment, and Disposal Scenarios

The experimental results using model food waste provide the technical foundation for the sustainability analysis, which models transportation of real university FW to existing paper mill AD facilities based on their geographic colocation. To analyze the relative sustainability of codigesting FW and PPMS, where the FW generated in universities is transported to paper mill ADs, we considered three baseline scenarios (Figures S2 and S3). Baseline scenario (i) models the disposal of FW from universities to landfills, which is the most typical disposal route for FW. Baseline scenario (ii) focuses on the anaerobic digestion of PPMS including the end-of-life disposal of digestate to a landfill. No pretreatment with PPMS was considered during this analysis. Lastly, baseline scenario (iii) models the anaerobic codigestion of FW and PPMS with end-of-life digestate disposal to a landfill. Since pulp and paper mills use anaerobic digestion to treat their waste and this waste must be disposed of, the FW generated at the universities is the only waste that is diverted from landfills in this model. The capital cost for the construction of a digestion plant is excluded from this analysis. To keep the model analysis consistent with the experimental part, only the amount needed to maintain a 1:1 ratio of FW to PPMS on a VS basis was considered. Pulp and paper mills generating more sludge than this were not included in this study. The temperature considered for the general analysis was 20 °C. Python^59^ (v3.12.4) was used for the design, simulation, sustainability assessment, and uncertainty and sensitivity analysis for all three baseline scenarios using quantitative sustainable design methodology.^60,61^ The code is publicly accessible on GitHub.^62^ In this analysis, we created three baseline scenarios and parallelly analyzed associated costs and environmental impacts. Given the limitations of the available data, a ± 10 to 25% variation was applied to assumed values (Table S1).

#### 2.2.2 Life Cycle Assessment

Life cycle assessment focused on the GWP (i.e., greenhouse gas (GHG) emissions as kg CO_2_ equivalents) estimation for all three scenarios. The analysis considered different emission sources: Scenario 1 included transportation and direct emissions from landfills; Scenario 2 covered transportation, energy, and direct landfill emissions; and Scenario 3 encompassed transportation, energy, materials, and direct landfill emissions. The Ecoinvent v3.6 database^63^ and the U.S. EPA’s tool for the Reduction and Assessment of Chemicals and Other Environmental Impacts, TRACI 2.1 v1.03 ^64^ were used to calculate the electricity-related impacts. For scenario 1, transportation emissions were based on FW transport to landfills, while direct emissions from landfills were computed using US EPA’s first-order kinetic model of methane production U.S. EPA.^65^ The model assumed that 25% of the produced methane is released into the atmosphere, with the remaining 75% captured and burned down. Scenario 2 transportation emissions accounted for the transportation of end-of-life sludge to landfills. Energy emissions were estimated based on the energy required to operate the AD reactor, and the direct landfill emissions were calculated using the first-order kinetics model. Scenario 3 transportation emissions considered FW transport to pulp and paper mills and subsequent end-of-life sludge disposal to landfills. Energy emissions were calculated for the energy needed to operate AD, and material emissions accounted for added sodium hydroxide. Direct landfill emissions for disposed sludge were determined using the first-order kinetics model. Electricity production emissions vary across states due to different energy sources. An average percentage of sources and their respective GWP was used to calculate GHG emissions (kg CO_2_ eq·tonne^–1^·day^–1^) from the model (Table S1).

#### 2.2.3 Techno-Economic Analysis

An economic analysis was performed to assess the cost for each scenario in USD per tonne of waste per day. The categories considered are transportation costs (wages and fuel costs), tipping fees, material costs, and AD energy consumption expenses. All costs were calculated at the present value. The analysis leverages existing anaerobic digestion facilities at pulp and paper mills, which typically operate AD systems for wastewater and sludge treatment. Codigestion with food waste utilizes available capacity in these established facilities, avoiding the capital costs associated with constructing a new treatment infrastructure. Different costs taken into consideration for each scenario were: scenario (i) accounted for transportation costs and tipping fees; scenario (ii) included transportation costs, tipping fees, and AD operation costs; and scenario (ii) included transportation costs, tipping fees, AD operation costs, and material costs. For scenario (i), transportation costs and tipping fees were estimated for moving and dumping the FW into landfills, respectively. For scenario (ii), transportation costs were calculated for moving the end-of-life PPMS to landfills, and tipping fees were calculated for dumping it. AD operating costs were calculated for the energy needed to operate the AD reactor. For scenario (iii), transportation costs were estimated for moving both FW in pulp and paper mills and end-of-life transportation of AD sludge to landfills. Transportation costs include both fuel costs (based on distance and fuel efficiency) and driver wages (based on travel times and hourly rates). These costs vary significantly based on the distance between waste generation sites and treatment/disposal facilities. Wages were calculated based on work hours and hourly pay, and tipping fees were calculated for the sludge disposal to landfills. Materials costs were estimated for sodium hydroxide added to the reactor to increase FW pH. Detailed modeling procedures for cost are described in the Supporting Information (Table S1).

#### 2.2.4 Contextual Analysis

To assess how the context affects economic and environmental outcomes for the combined treatment scenario, the states in the US were grouped into six regions (Northeast, Southeast, Midwest, South Central, Mountain Plains, and Pacific). These regions were selected based on different geographical locations, and general assumptions were changed to reflect the representative condition in specific regions. Specifically, we used region-specific data on temperature,^66^ electricity prices, and electricity mixes (to calculate GHG emissions associated with energy requirements).^67^ University locations were obtained from the National Center for Education Statistics; paper mill locations were from EPA facility registries; and landfill locations were from the Waste Atlas database. Transportation distances were calculated using GIS and Google Maps analysis, assuming waste transport to the nearest appropriate facility for each scenario. Regional variations in fuel costs and driver wages were incorporated using state-specific data from the Bureau of Labor Statistics databases. The outcome of this analysis helps evaluate the performance of the system when it is deployed across a range of contexts by considering local conditions.

#### 2.2.5 Uncertainty and Sensitivity Analyses

For each uncertain parameter, distributions were defined, and an additional variability of up to 25% was added into unit cost and environmental factors to account for the variations in unit prices and impacts.^68,69^ This degree of uncertainty level is consistent with established practices in sustainability of waste treatment technologies, where this range accounts for variability in operational and market conditions when comprehensive site-specific data are limited.^65,66^ For all scenarios, Monte Carlo simulations with Latin hypercube sampling (10,000 samples) were carried out for uncertainty analysis.^70^ This process generates a distribution of results, from which median, fifth percentile, and 95th percentile values are presented in the findings. Subsequently, Spearman’s rank correlation coefficient was computed based on the input and output distributions from the simulation to evaluate the results’ sensitivity to individual variables. Sensitivity refers to the extent to which an output (i.e., costs and GHG emissions) is correlated with a single input parameter. To calculate Spearman’s rank correlation coefficients, values in each input and output were ranked (e.g., the lowest value is ranked 1, the second lowest ranked 2, and so on), and the correlation between these ranks was determined. A coefficient value representing the correlation shows the extent to which an arbitrary monotonic function can describe the relationship between the input parameter and the output result. Here, the coefficient values ranged from −1 to 1, with a large absolute value indicating a stronger correlation.

## 3 Results and Discussion

### 3.1 Substrate and Inoculum Characterization

Characterization results for both codigestion (FW-PPMS) and monodigestion (PPMS) systems were collected at the beginning and the end of the experiment (Table 1). The initial low pH of FW was adjusted with sodium hydroxide to maintain a suitable range (6.5–7.6) for the microbes.^71^ There was a small increase in pH for both systems, mainly due to the reduction in volatile fatty acids over time as VFAs are converted to acetate in the acetogenesis step.^72^ The final CODs for the codigestion and monodigestion systems were 431 and 450.2 mg/L, respectively. A 92% COD reduction was achieved in the codigestion system, on par with a similar time-scale study that found 87% soluble COD removal, suggesting an improved sludge treatment performance.^25^ On the other hand, the COD reduction in the monodigestion system was 80.5%. The codigested system had a C/N molar ratio of 0.38 at the start of the experiment, and this ratio decreased to 0.15 by the end. In contrast, the monodigestion system began with a C/N ratio of 0.11 and increased to 0.16 by the end of the process. The C/N decrease in the codigested system suggested an improved carbon consumption due to the availability of nutrients supporting microbial growth.

**Table 1:** Characterization and Analytical Results of Substrate and Inoculum for Anaerobic Codigestion Experiments^a^

	**FW-PPMS**			**PPMS**		
**parameters**	**beginning**	**end**	**% difference (absolute)**	**beginning**	**end**	**% difference (absolute)**
pH	6.7 ± 0.1	7.3 ± 0.1	8.6 ± 0.24	7.4 ± 0.1	7.5 ± 0.2	1.3 ± 0.05
Alkalinity (mg/L as CaCO_3_)	2103.1 ± 91.0	2959.7 ± 272.1	33.8 ± 4.57	2184.0 ± 79.3	2566.8 ± 151.2	16.2 ± 1.54
TS (g/L)	21.0 ± 1.7	16.8 ± 2.7	22.2 ± 5.36	25.3 ± 1.5	21.0 ± 2.9	18.4 ± 3.63
VS (% of TS)	9.2 ± 2.6	6.5 ± 0.7	34.2 ± 13.35	7.5 ± 2.9	6.2 ± 1.3	19.1 ± 11.4
VFA (mg/L CH_3_–COOH)	1320.0 ± 231.3	307.2 ± 82.5	124.5 ± 55.25	459.0 ± 54.0	246.0 ± 23.4	60.4 ± 12.85
COD (mg/L)	5379.2 ± 221.8	431.0 ± 19.9	170.3 ± 14.88	2310.8 ± 54.5	450.2 ± 51.5	134.8 ± 18.60
TOC (mg/L)	350.6 ± 14.6	51.9 ± 2.5	148.4 ± 13.33	179.9 ± 8.7	54.8 ± 4.5	106.6 ± 13.91
TN (mg/L)	921.4 ± 117.8	353.9 ± 10.5	89.0 ± 14	1672.1 ± 88.4	338.1 ± 19.4	132.7 ± 14.63
NH_3_–N (mg/L)	0.4 ± 0.1	0.2 ± 0.1	56.3 ± 42.22	0.3 ± 0.0	0.2 ± 0.0	38.6 ± 0

a Key parameters were measured at the beginning and end of the experimental period for both the food waste and pulp and paper mill sludge (FW-PPMS) codigestion system and the PPMS monodigestion system. Parameters include pH, total solids (TS), volatile solids (VS), chemical oxygen demand (COD), volatile fatty acids (VFA), alkalinity, ammonia nitrogen (NH_3_–N), total organic carbon (TOC), and total nitrogen (TN). Values are expressed as average ± the standard deviation. These measurements provide insight into the bioreactor environment and the efficiency of organic matter degradation during the anaerobic digestion process.

The higher COD reduction (92 vs 80.5%) in the codigestion system can potentially be attributed to the higher nutrient availability and diverse microbial community supported by the cosubstrate mixture (which is explored in Section 3.3).^41^ Consistency in pH values within the optimal range for both systems, despite the acidic nature of FW, demonstrated effective buffering capacity, crucial for stable methanogenesis.^73^ The reduction in VFAs over time, coupled with the maintained alkalinity, suggests successful progression through the various stages of anaerobic digestion, particularly the conversion of VFAs to acetate and ultimately to methane. The changes in TS, VS, and NH_3_–N concentrations provide further evidence of organic matter mineralization and nutrient assimilation during the digestion process. Overall, these results suggest that codigestion of FW with PPMS not only improved the efficiency of the anaerobic digestion process but also potentially led to more stable operation compared to PPMS monodigestion.

### 3.2 Biogas Production from the Biochemical Methane Potential (BMP) Experiment

The reactors were operated for 31 days, with monodigestion reactors producing gas for 20 days and codigestion ones for 28 days (Table S2). At the end of the experimental period, specific methane yields found for the monodigestion were 111 mL CH_4_/g VS and 151 mL CH_4_/g VS for codigestion, resulting in a 36% increased methane production in the codigestion system (Figure 1a). Statistical analysis using a *t* test confirmed this difference was significant (*p* = 0.025, *n* = 4 for codigestion, *n* = 3 for monodigestion). While the FW-PPMS system had higher initial COD due to food waste addition, the 36% increase in methane yield cannot be attributed solely to higher COD content as yields were normalized to volatile solids and reflect improved biodegradability and synergistic effects between substrates. The 36% increase in specific methane yield in the codigestion system represents a substantial improvement in treatment, suggesting an enhanced microbial activity and a more complete organic substrate utilization. The extended gas production period for codigestion (28 days versus 20 days for monodigestion) suggests a more sustained and efficient degradation process, likely due to an increase in the concentration of readily biodegradable organic matter and nutrients provided by the FW, which was capable of sustaining a more diverse microbial community. Supporting a stable anaerobic process, promoted by the FW readily available organic substrate, results in a higher potential for recalcitrant organic matter degradation (cellulose and lignin in this case), resulting in lower effluent COD and higher methane production. The overall performance of the codigestion system was also due to a higher carbon-to-nitrogen ratio, improved nutrient availability, and synergistic effects between the microbial communities degrading the two substrates. These findings highlight the potential of codigestion as a strategy to optimize biogas production from PPMS while simultaneously addressing FW management challenges, offering a dual solution for these waste streams that could lead to both environmental and economic benefits in full-scale applications.

**Figure 1:**
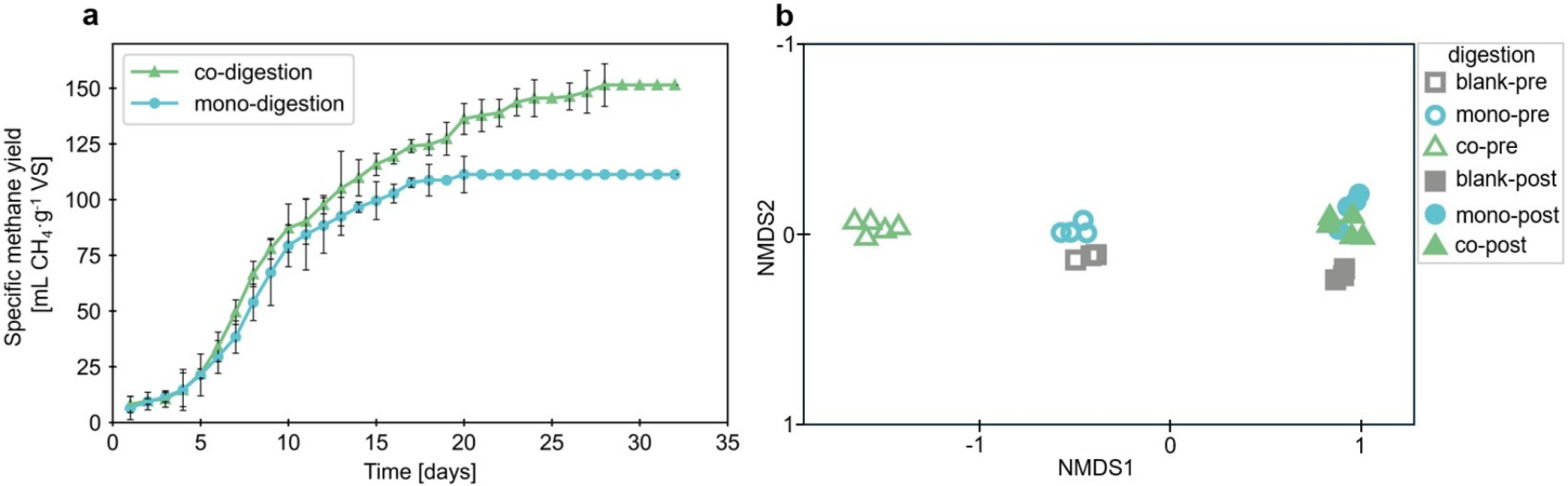
(a) Comparison of cumulative specific methane yields from anaerobic codigestion of food waste and pulp and paper mill sludge (FW-PPMS) and monodigestion of pulp and paper mill sludge (PPMS) over a 31-day time period. Methane production from the blank reactors was subtracted during the specific methane yield calculation for both codigestion and monodigestion. Error bars indicate the standard deviation of measurements from digesters set up in quadruplicate (codigestion) and triplicate (monodigestion), providing insight into the variability and reproducibility of the results. (b) Nonmetric multidimensional scaling (NMDS) ordination of genus counts for three different anaerobic digestions (blank, monodigestion, and codigestion) at the beginning (pre) and end (post) of the experimental period. Data was transformed to square root for resemblance analysis and the 2D stress was 0.03.

### 3.3 Microbial Community Analyses

To assess the microbial community health and diversity, DNA was extracted from each reactor from the BMP experiments and sequenced by utilizing long-read 341-1492 PCR primers with a barcode on the forward end. A total of 224 genera were identified throughout all of the samples. A statistical analysis comparing the composition of the bacterial community among digestions was conducted on the whole microbial community, regardless of abundance. The genera *Trichococcus*, *Clostridium*, and *Peptostreptococcus* were the three most abundant genera in all the predigestion except for the codigestion which had *Enterococcus* as the second most abundant, *Clostridium* as the third most abundant, and *Peptostreptococcus* as the fourth (Figures S4, S5, and S6). These were mainly responsible for breaking down the higher molecular weight organic matter and carrying out the fermentation process. Of these genera, *Clostridium* was the most abundant in all the post-digestion, whereas *Peptostreptococcus* and *Enterococcus* were not abundant in any. *Trichococcus* was only found to be abundant in the post-codigestion where it decreased from 78.7% of the total in the pre to 1.3% in the post (Figure S4). PERMANOVA suggested differences in the community composition among the different reactors (*p* = 0.001) with nonmetric multidimensional scaling (NMDS) indicating that much of this variation was being driven by pre-codigestion samples (Figure 1b). Monodigestion and blank presamples clustered close together indicating similarity while all the post-digestion clustered together and were separate from any of the predigestion. All three of the post-digestion results clustering together in the NMDS suggested that, regardless of the initial inputs, the microbial communities became more uniform across all reactors toward the end. This pattern is also reflected in the diversity calculations. Codigestion presamples showed less diversity and evenness than monodigestion and blank presamples (Table S3). After going through the bioreactors, the diversity and evenness of codigestion increased, whereas, for the other two digestions, it decreased slightly. Although genus-level taxonomic identification does not guarantee specific metabolic functions, the substantial apparent increases in diversity indices for codigestion (Shannon: ∼150% increase; Simpson: ∼142% increase) suggest meaningful changes in microbial community structure. However, future studies should incorporate larger sample sizes and include functional gene analysis or enzyme assays to provide more definitive evidence of metabolic capabilities and the statistical validation of diversity changes. Overall, these findings align with previous studies reporting similar microbial community convergence during anaerobic codigestion of FW and PPMS.^29,43^

For a comparison of metabolic activities of the genera, we focused on abundant genera in each replicate. Metabolic capabilities of the abundant genera (carbohydrates, proteins) were compared among pre- and post-digestions. PERMANOVA suggests no differences in the metabolic capabilities of abundant genera among any of the digestions (*p* = 0.937). This is supported by the NMDS which shows that apart from the monodigestion post samples, most of the samples clustered relatively close together with a few scattered points (Figure S7). Although the small sample size makes it difficult to draw definitive conclusions, the similarities in the microbial communities in all the post-digestions compared to the variation in the different starting treatments, suggest that the AD process itself may drive community convergence regardless of initial substrate composition.^74^

### 3.4 Financial Viability and Environmental Implications

Three distinct scenarios were compared to evaluate the potential advantages of integrating food waste management with industrial byproduct treatment, particularly through codigestion, against more traditional disposal methods (Figure 2). The first scenario represents the FW disposal directly in landfills. The second scenario focuses on the treatment of PPMS through AD, followed by the end-of-life disposal of the resulting digestate in landfills. The third scenario introduces an innovative approach where FW and PPMS are combined and codigested, with the digestate ultimately disposed of in landfills. For the first scenario, the daily treatment cost per tonne of FW was 405.13 USD·tonne^–1^·day^–1^ (median) with a range of 223.54-913.37 USD·tonne^–1^·day^–1^ [hereinafter, fifth −95th percentiles are shown in brackets]. The PPMS treatment scenario cost was 144.20 [95.35–231.50] USD·tonne^–1^·day^–1^, and the combined scenario was projected to cost 236.64 [126.67–431.71] USD·tonne^–1^·day^–1^. The higher cost of FW can primarily be attributed to the higher tipping fees and transportation costs. For the PPMS and combined scenarios, the transport and other expenses are offset by energy production from methane gas collected during the AD process. Emissions from the FW scenario are the highest at 556.27 [427.07–903.89] kg CO_2_ eq·tonne^–1^·day^–1^, compared to 140.23 [81.36–231.11] kg CO_2_ eq·tonne^–1^·day^–1^ for PPMS, and 228.30 [81.12–581.26] kg CO_2_ eq·tonne^–1^·day^–1^ for combined scenarios. The high variability in emissions results for the combined scenario is due to the larger range of transportation distance between universities and paper mills. The higher emissions from the FW scenario can mainly be attributed to the transportation emissions and huge amounts of methane produced at landfills that directly go into the atmosphere. In the PPMS and combined scenarios, the produced methene is converted to energy, reducing the overall GWP of the system. ANOVA testing was further performed to evaluate the statistical significance of cost and GWP results for different scenarios. The ANOVA results proved that the costs and GWP values are significantly different with *p*-values >0.001 and *F* < Fcrit (Tables S3 and S4).

**Figure 2:**
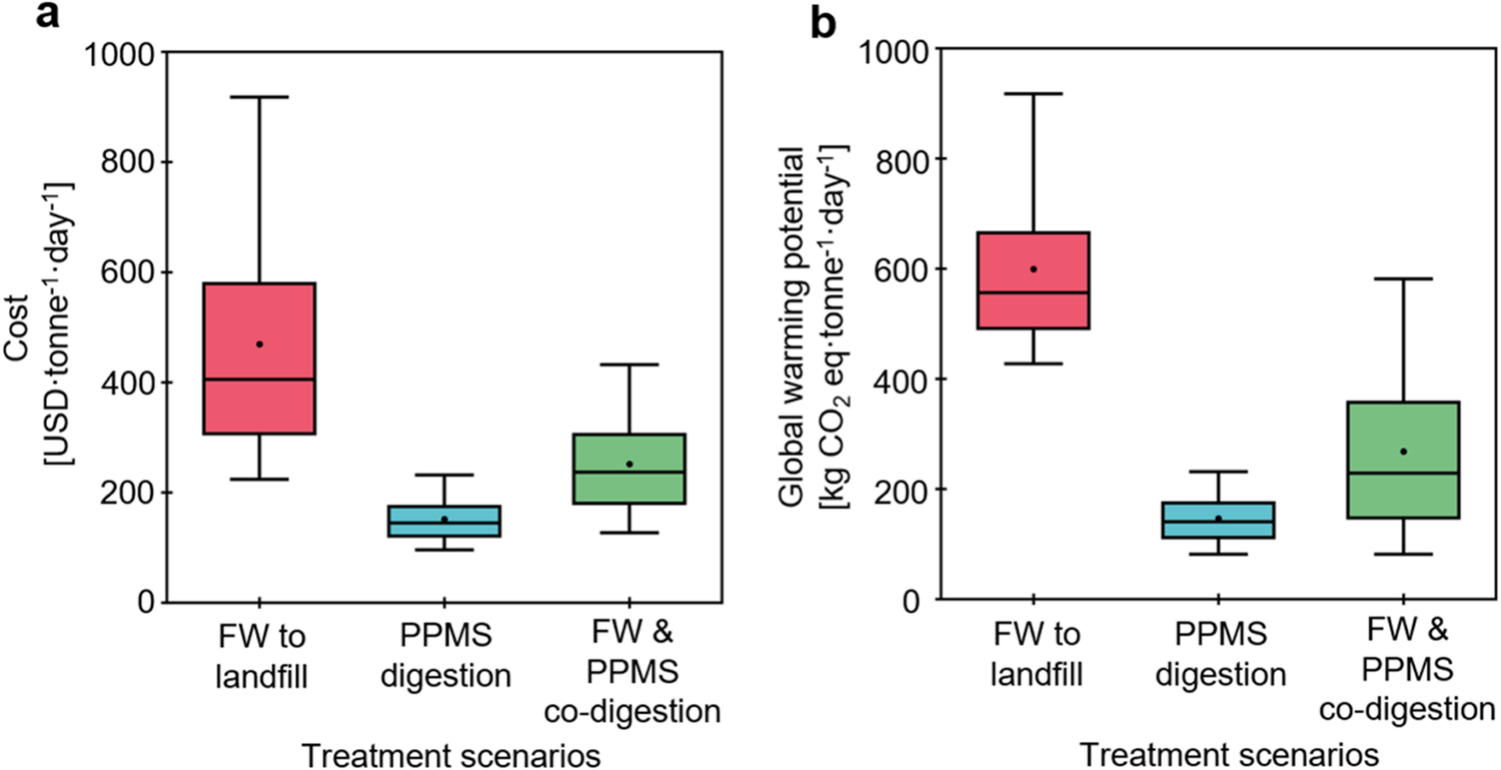
Estimated costs (a) and global warming potential (b) for three baseline scenarios: food waste (FW) to landfill, treatment of pulp and paper mill sludge (PPMS) including end-of-life disposal to landfill, and combined treatment including end-of-life disposal to landfill. Box and whiskers show the median values (center line), mean values (point), 25th and 75th percentiles (bottom and top of the box), and 5th and 95th percentiles (lower and upper whiskers) from the uncertainty analysis of 10,000 Monte Carlo simulations.

The analysis of these three scenarios revealed important insights into the economic and environmental implications of different waste treatment approaches. Looking at the median values, it is evident that the combined treatment scenario emerges as a particularly promising option, striking a balance between cost-effectiveness and environmental sustainability. While it is a little bit more expensive than treating PPMS alone, it offers significant cost savings compared to handling FW separately, while also providing substantial environmental benefits. The dramatic reduction in greenhouse gas emissions for the PPMS and combined scenarios compared to the FW scenario underscores the environmental advantages of anaerobic digestion over landfilling. By capture and utilization of methane for heat production, these approaches effectively mitigate a major source of greenhouse gas emissions associated with waste management. This aligns with broader goals of reducing the carbon footprint of waste treatment processes and moving toward more circular economy practices. Moreover, the economic viability of the combined scenario, coupled with its environmental benefits, suggests that the codigestion of FW with PPMS could be an advantageous solution for waste managers and environmental policymakers. It offers a pathway to address the challenges of FW management while simultaneously improving the efficiency and sustainability of PPMS treatment. This integrated approach exemplifies how synergies between different waste streams can be leveraged to create more sustainable and economically viable waste management systems.

### 3.5 Elucidating Drivers for Cost and Emission

The next phase of our study was to elucidate the key drivers influencing costs and emissions across all three scenarios (Figure 3). The categories for cost included transportation, tipping fees, operation, and materials. Emission categories covered operation, transportation, energy, materials, and direct landfill emissions. In FW scenarios, the moisture content, landfill distance, and tipping fees are the primary drivers contributing to costs. Moisture content directly affects FW mass, impacting tipping fees and transportation costs. The median costs for the FW scenario are 125.57 and 504.54 USD·tonne^–1^·day^–1^ for FW moisture content of 20 and 80%, respectively (Figure S8a). Other key factors are average speed and driver wages, both directly affecting transportation costs. For the PPMS scenario, landfill distance, average speed, and driver wages have significant impacts that contribute to transportation-related costs. Other notable factors include tipping fees, reactor diameters, and electricity cost. Moisture content and the amount of PPMS are two important factors affecting the tipping fees. In the combined system, the transportation distance has the greatest impact on costs. Other key factors include tipping fees, average speed, driver wages, and landfill distance. The moisture contents of both FW and PPMS also notably affect overall costs.

**Figure 3:**
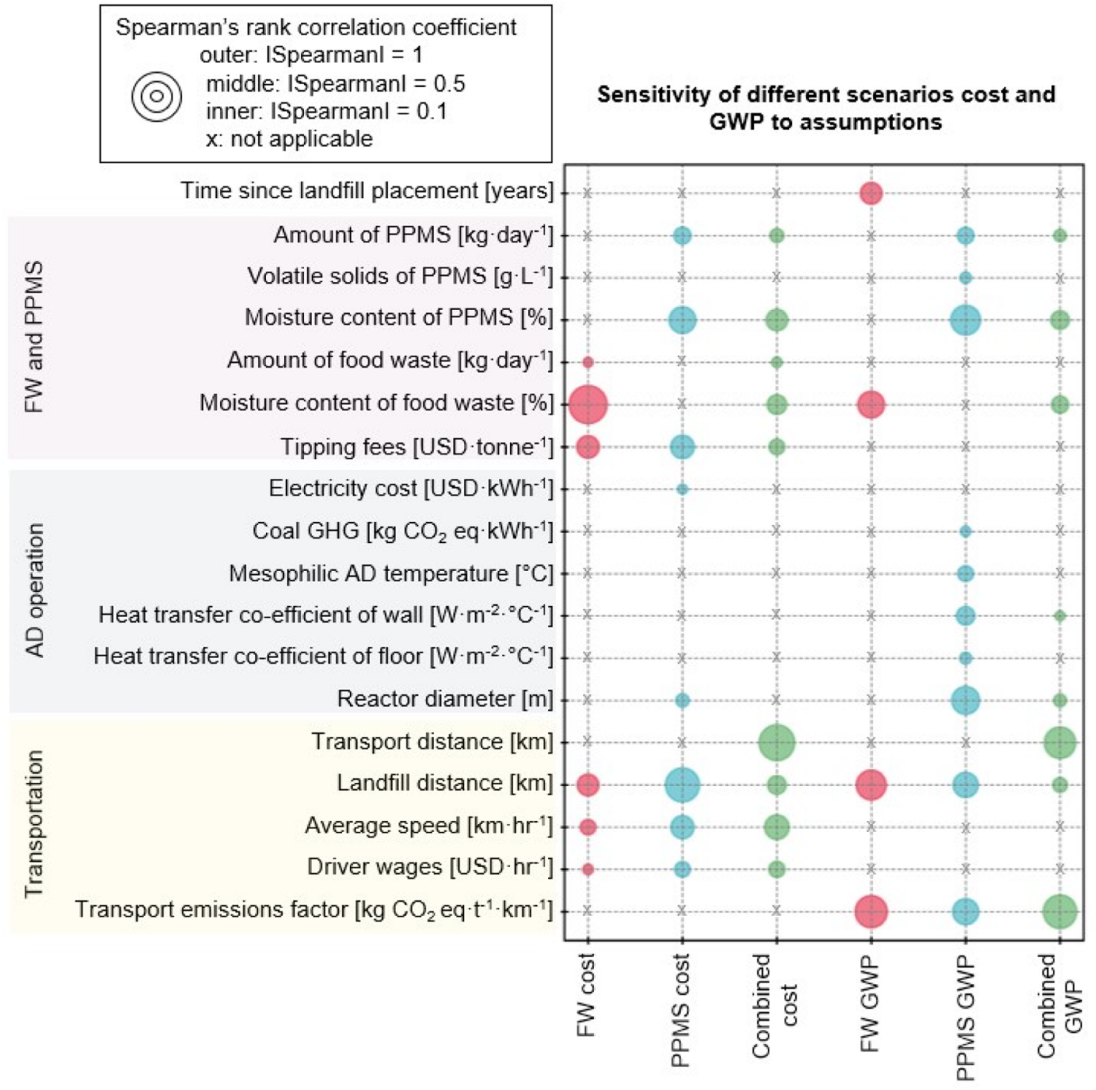
Spearman’s rank correlation coefficients for the daily cost and greenhouse gas (GHG) emission per tonne of waste for the three baseline scenarios (food waste to landfill, treatment of pulp and paper mill sludge including end-of-life disposal to landfill, and combined treatment including end-of-life disposal to landfill).

Regarding GWP drivers for the FW scenario, the most influencing factors are moisture content, landfill age, transport emission factor, and landfill distance. Landfill age is considered since the initial refuse placement in a landfill where longer periods result in higher methane production and release into the atmosphere. Landfill distance and transport emission factors directly impact GWP through transportation emissions. In the PPMS scenario, moisture content has the greatest impact, as it directly influences solid content and methane production. Similar to the FW scenario, landfill distance and transport emission factor are two key drivers influencing the emissions. Other notable factors include the amount of PPMS, reactor diameter, volatile solids content, reactor temperature, and coal GHG. The heat transfer coefficients of the floor and wall are also two important drivers impacting operation-related costs. For the combined scenario, the transport emissions factor and transport distance have the most significant impact. This is because the transportation distance plays a crucial role in moving FW to the pulp and paper mill industry. The median GWP for the combined treatment scenario is 84.93 and 415.09 kg CO_2_ eq·tonne^–1^·day^–1^ for transportation distances of 6 km and 400 km, respectively (Figure S9b). Additional key factors include the moisture content of FW and PPMS, landfill distance, amount of PPMS, and reactor diameter.

### 3.6 Financial Viability and Environmental Implications Across Contexts

To understand the impact of context, we evaluated the financial viability and environmental implications of six regions in the US (Figure 4). Key factors influencing these outcomes include temperature, distances between universities and paper mills and between paper mills and landfills, driver wages, tipping fees, and GWP for heat production. All regions show variations in cost and GWP. The Mountain Plains region has the highest median cost at 523.96 USD·tonne^–1^·day^–1^ and emissions at 744.45 kg of CO_2_ eq·tonne^–1^·day^–1^. The primary reason for this is the limited number of universities and pulp and paper mills located in that region and the long distances between them (Figure S1). The region’s low temperature also requires more electricity for anaerobic digestion, increasing costs and emissions. The Northeast has the lowest median cost (205.57 USD·tonne^–1^·day^–1^) and the lowest median emissions (203.52 kg of CO_2_ eq·tonne^–1^·day^–1^). The high concentration of universities and paper mills in these regions that directly impacts transportation-related costs and emissions can primarily be attributed to this. Other regions show higher costs and emissions due to fewer universities and paper mills, and the heat needed to run the AD system.

**Figure 4:**
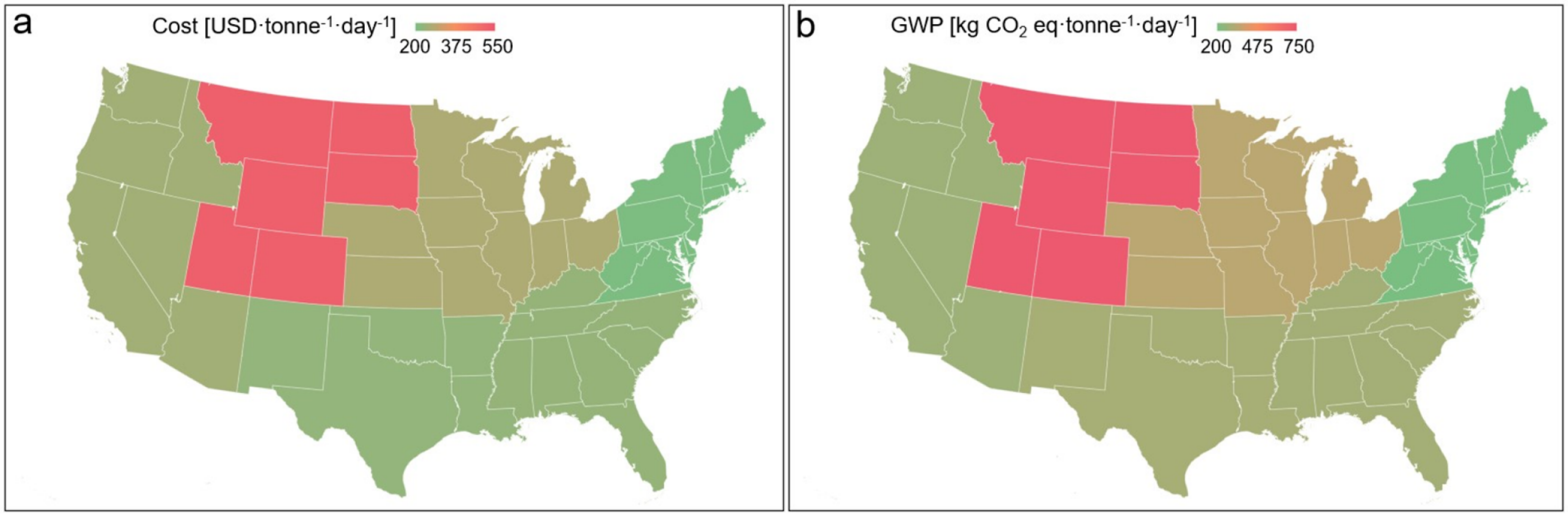
States in the US divided into six regions (Northeast, Southeast, Midwest, South Central, Mountain Plains, and Pacific), and the map showing the potential reduction in costs (a) and GWP (b) in each region.

This regional analysis highlights the significant impact of geographical and infrastructural factors on the economic and environmental performance of the combined treatment scenario. The stark contrast between the Mountain Plains region and the Northeast region underscores how the density of facilities, transportation distances, and climate can dramatically influence both costs and emissions. These findings emphasize the importance of considering local context in waste management planning, suggesting that the viability and sustainability of codigestion systems may vary considerably across different parts of the country. The results also point to potential strategies for optimizing these systems, such as prioritizing implementation in areas with high facility density and favorable climates or exploring ways to reduce transportation distances and energy requirements in less favorable regions. This nuanced understanding of regional variations can inform more targeted and effective policies and investments in sustainable waste management infrastructure across the US.

## 4 Conclusions

Higher COD removal and methane yields in codigested bench-scaled experiments highlighted the effective synergy of the two FW and PPMS feedstocks. The increased methane production also ensured higher volatile content removal, resulting in improved sludge treatment and quality. Microbial analysis results for the codigestion showed that the microorganism community was more uniform toward the end of the experimental period than at the beginning. A more uniform microbial community adapted to the different feedstocks has greater potential for better sludge COD removal and methane production. Under the general set of assumptions (without any local context consideration), the cost analysis results showed that processing FW separately is costlier than handling it with PPMS, indicating the advantages of a codigestion approach. While managing PPMS alone is cheaper than combining it with FW, the latter significantly reduces the costs of managing FW alone. On the other hand, the emissions are higher for handling FW alone compared to the other two scenarios. The high emissions come from FW transportation to landfills and later direct landfill emissions. The combined treatment scenario has much lower emissions compared to the FW scenario, although it is slightly higher than treating PPMS alone. Thus, the combined treatment of FW-PPMS can substantially reduce costs and emissions compared to handling FW independently while improving the digestibility of PPMS. Analyzing costs and environmental impacts across contexts revealed the feasibility of implementing this in different locations. The Mountain Plains region has the highest cost and emissions, whereas the Northeast region has the lowest, making the latter more favorable for adopting the codigestion system. Regions like the Mountain Plains might need to explore other feasible ways to reduce costs and emissions. This contextual analysis can be adjusted to include more localized parameters to any specific paper mill with an available capacity in the future. It should be noted that this study has some limitations that might impact the interpretation of the results. The BMP results might not be fully representative when used on a large scale with varying FW, potentially affecting the methane production from the system. Thus, future studies can be conducted with diverse types of FW to evaluate the performance of the codigestion system at different scales. Additionally, the retrofitting costs of leveraging paper mills’ AD facilities need a clear understanding. Also, the impact of other contaminants in paper mill sludge, such as PFAS,^75^ can be further explored in the codigestion system. In conclusion, the results emphasize the potential of codigestion to improve waste management both economically and environmentally.

## Notes

Inputs for life cycle assessment units and parameters (Table S1); daily methane production data from codigestion and monodigestion experiments over 31 days (Table S2); Shannon-Weiner and Simpson diversity metrics for microbial communities (Table S3); ANOVA results for cost and global warming potential analyses of baseline scenarios (Tables S4 and S5); colocation map of paper mills and large universities in the United States (Figure S1); process flow diagrams for three baseline scenarios and codigestion system (Figures S2 and S3); microbial community composition showing abundant genera percentages in pre- and post-digestion samples for codigestion, monodigestion, and blank treatments (Figures S4–S6); nonmetric multidimensional scaling ordination of metabolic profiles (Figure S7); sensitivity analyses for moisture content and transportation distance effects on cost and global warming potential (Figures S8 and S9); specific methane yield calculation methodology (Section S1); paper mill treatment process description (Section S2); and detailed microbial community analysis methods including DNA extraction, PCR amplification, sequencing, and statistical analyses (Section S3) (PDF)
Design parameters for sustainability analysis
